# RETRACTION: MCCK1 enhances the anticancer effect of temozolomide in attenuating the invasion, migration and epithelial‐mesenchymal transition of glioblastoma cells in vitro and in vivo

**DOI:** 10.1002/cam4.70300

**Published:** 2024-10-10

## Abstract

**Retraction:** T. Liu, A. Li, Y. Xu, and Y. Xin, “MCCK1 Enhances the Anticancer Effect of Temozolomide in Attenuating the Invasion, Migration and Epithelial‐Mesenchymal Transition of Glioblastoma Cells in Vitro and in Vivo,” *Cancer Medicine* 8, no. 2 (2019): 751–760. https://doi.org/10.1002/cam4.1951.

The above article, published online on 18 January 2019, in Wiley Online Library (wileyonlinelibrary.com), has been retracted by agreement between the authors, the journal Editor‐in‐Chief, Stephen Tait, and John Wiley & Sons Ltd. A report submitted by a third party noted image duplications with other articles by different author groups. Specifically, the report found image duplications with other published articles in Figures 1E, 2B, 3A, 3D and 4C. The report also found image overlap between images in Figure 3A. The retraction has been agreed to because the evidence of image duplication across different articles, each of which describes different experimental conditions, fundamentally compromises the editors' confidence in the conclusions presented. The authors have voluntarily agreed with the retraction.

**Retraction:** T. Liu, A. Li, Y. Xu, and Y. Xin, “MCCK1 Enhances the Anticancer Effect of Temozolomide in Attenuating the Invasion, Migration and Epithelial‐Mesenchymal Transition of Glioblastoma Cells in Vitro and in Vivo,” *Cancer Medicine* 8, no. 2 (2019): 751–760. https://doi.org/10.1002/cam4.1951.

The above article, published online on 18 January 2019, in Wiley Online Library (wileyonlinelibrary.com), has been retracted by agreement between the authors, the journal Editor‐in‐Chief, Stephen Tait, and John Wiley & Sons Ltd. A report submitted by a third party noted image duplications with other articles by different author groups. Specifically, the report found image duplications with other published articles in Figures 1E, 2B, 3A, 3D and 4C. The report also found image overlap between images in Figure 3A. The retraction has been agreed to because the evidence of image duplication across different articles, each of which describes different experimental conditions, fundamentally compromises the editors' confidence in the conclusions presented. The authors have voluntarily agreed with the retraction.

